# SFRS7-Mediated Splicing of Tau Exon 10 Is Directly Regulated by STOX1A in Glial Cells

**DOI:** 10.1371/journal.pone.0021994

**Published:** 2011-07-06

**Authors:** Daan van Abel, Dennis R. Hölzel, Shushant Jain, Fiona M. F. Lun, Yama W. L. Zheng, Eric Z. Chen, Hao Sun, Rossa W. K. Chiu, Y. M. Dennis Lo, Marie van Dijk, Cees B. M. Oudejans

**Affiliations:** 1 Department of Clinical Chemistry, VU University Medical Center, Amsterdam, The Netherlands; 2 Department of Clinical Genetics, Section Medical Genomics, VU University Medical Center, Amsterdam, The Netherlands; 3 Department of Chemical Pathology, Prince of Wales Hospital, Shatin, New Territories, Hong Kong SAR, China; McGill University, Canada

## Abstract

**Background:**

In this study, we performed a genome-wide search for effector genes bound by STOX1A, a winged helix transcription factor recently demonstrated to be involved in late onset Alzheimer's disease and affecting the amyloid processing pathway.

**Methodology/Principal Findings:**

Our results show that out of 218 genes bound by STOX1A as identified by chromatin-immunoprecipitation followed by sequencing (ChIP-Seq), the serine/arginine-rich splicing factor 7 (SFRS7) was found to be induced, both at the mRNA and protein levels, by STOX1A after stable transfection in glial cells. The increase in SFRS7 was followed by an increase in the 4R/3R ratios of the microtubule-associated protein tau (MAPT) by differential exon 10 splicing. Secondly, STOX1A also induced expression of total tau both at the mRNA and protein levels. Upregulation of total tau expression (SFRS7-independent) and tau exon 10 splicing (SFRS7-dependent), as shown in this study to be both affected by STOX1A, is known to have implications in neurodegeneration.

**Conclusions:**

Our data further supports the functional importance and central role of STOX1A in neurodegeneration.

## Introduction

Storkhead box 1 (STOX1A), a transcription factor structurally and functionally related to the forkhead family of transcription factors, characterized by a 100 amino acid DNA-binding motif termed the winged helix domain, was recently found to be a susceptibility gene for pre-eclampsia [1], [2], a hypertensive disorder of pregnancy which remains a major cause of maternal and perinatal mortality and morbidity [3]. Paradoxically, STOX1A was also found to be functionally involved in late-onset Alzheimer's disease (LOAD) [4], a progressive neurodegenerative disease of the brain which is characterized by memory loss and impaired visiospatial skills involving elderly patients (*>*65 years) of both sexes [6]. In the latter study, van Dijk and co-workers showed that STOX1A is expressed abundantly in the brain, correlates with the severity of late onset Alzheimer's disease (LOAD) and transactivates the leucine-rich repeat transmembrane 3 (*LRRTM3*) gene [4]. Upregulation of the *LRRTM3* gene by STOX1A leads to increased amyloid-*β* precursor protein (APP) processing resulting in higher levels of amyloid β peptide [4]. Amyloid β deposition is a key event in the etiology of Alzheimer's disease (AD) [5], [6]. However, STOX1A transactivation of *LRRTM3* could not explain the complete STOX1A expression profile identified in the Alzheimer brain. Given the importance of this finding in neurodegeneration, but the lack of insight in the number and nature of genes controlled by STOX1A in the brain, we started a systematic search for downstream effector genes in the brain. For this, we performed a genome-wide search for STOX1A binding sites in the neuroblastoma cell-line SK-N-SH by chromatin-immunoprecipitation followed by sequencing (ChIP-Seq) [7]. Subsequently, genes selected for their involvement in pathways leading to LOAD and other neurodegenerative diseases were explored in detail.

## Results

### High throughput sequencing identifies a STOX1A DNA binding region in the promoter of SFRS7, a gene involved in the splicing of tau exon 10

Using a genome-wide, antibody-independent approach, 218 genomic regions were found to be bound by STOX1A in SK-N-SH cells (**Dataset S1**). With the algorithm Cis-regulatory Element Annotation System (CEAS) we filtered for known functionally important genomic regions [8]. This resulted in a top list of 115 hits associated with their corresponding regulated genes (**Dataset S2)**. Out of 13 genes directly positioned in known promoter elements (**Table 1**), we selected the serine/arginine splicing factor 7 (SFRS7) for in-depth functional analysis given its proven effect on tau exon 10 splicing [9], [17]. Misregulation of tau exon 10 splicing has a pathogenic role in neurodegenerative diseases [10], [11].

**Table 1:** STOX1A regulated genes identified using ChIP sequencing.

Gene symbol	Name
RAB10	ras-related GTP-binding protein
AHCY	S-adenosylhomocysteine hydrolase
FSIP	fibrous sheath interacting protein 1
RPL17	ribosomal protein L17
SFRS7	splicing factor, arginine/serine-rich 7
CALM2	calmodulin 2
CHST12	carbohydrate (chondroitin 4) sulfotransferase
WNT2B	wingless-type MMTV integration site family
CADD45B	Growth arrest and DNA-damage-inducible, beta
CLASP1	CLIP-associating protein 1
PSMF1	proteasome inhibitor subunit 1
ACTG1	actin, gamma 1 propeptide
FANK1	fibronectin type III and ankyrin repeat domains

CEAS annotation software was used to filter for regions of known functional importance for which 13 genes were positioned directly in known promoter regions.

### SFRS7 mRNA and protein expression levels are increased in SK-N-SH cells stably transfected with STOX1A

**Figure 1:**
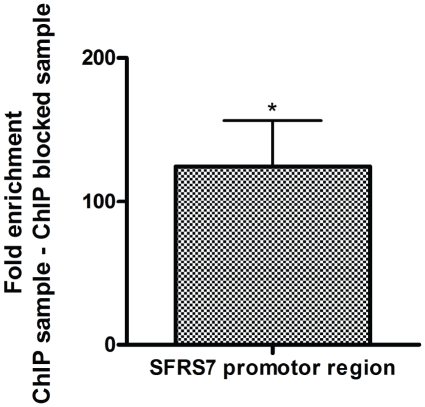
Validation by quantitative PCR of SFRS7 binding by STOX1A. Results show a mean 125 fold (mean ΔΔCt is −6,96) enrichment for SFRS7 in STOX1A stimulated ChIP DNA compared with their negative controls (ChIP sample vs ChIP blocked sample). Bars are mean ± SEM. Asterisks indicate *P*<0.05 (one sample t-test with theoretical mean 0). Data were obtained from at least five independent ChIP samples from which each DNA sample was measured in triplicate.

**Figure 2:**
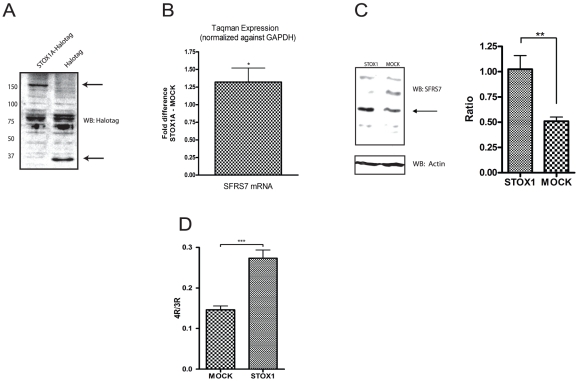
STOX1A-induced SFRS7 expression is followed by increased tau splicing in SK-N-SH cells. (A) Expression of STOX1A protein was determined with an anti-Halotag specific antibody by western blot using total cell protein extracts obtained from stable STOX1A (left lane) and MOCK (right lane) transfected SK-N-SH cells. A specific band representing STOX1A-halotag protein was observed at its expected size of 150 kd. Halotag protein (MOCK) was detected at its expected size of 34 kd. Westernblot image is a representative of at least 3 independent experiments. (B) Quantitative RT-PCR data shows a mean 1.32 fold (mean ΔΔCt is −0,4) increased mRNA expression for SFRS7. Bars are mean ± SEM. * indicate *P*<0.05 (one sample t-test with theoretical mean 0). N = 4, each sample was measured in triplicate. (C, left image) Expression of endogenous SFRS7 protein was determined with an SFRS7 specific antibody by western blot using total cell protein extracts obtained from stable STOX1A and MOCK transfected SK-N-SH cells. An antibody specific for actin was used as a loading control. (C, right graph) Quantification of SFRS7 protein was performed using densitometry. The ratio number of obtained band intensities for STOX1A divided by actin was compared to the ratio number of obtained band intensities for MOCK divided by actin for 3 independent experiments. A significant increase for the STOX1A ratio number was found compared to the MOCK ratio number. *P*-values were calculated using two-tailed unpaired *t*-test, error bars represent ± SEM, ** indicate *P*<0.01. (D) Ratio numbers were obtained by dividing endogenous 4R and 3R tau mRNA concentrations for SK-N-SH cells stably transfected with STOX1A or MOCK constructs. Endogenous 4R and 3R mRNA concentrations were obtained as described in the material and methods section. A highly significant increase of the 4R/3R tau ratio was seen for STOX1A compared to MOCK transfected cells. *P*-values were calculated using two-tailed unpaired *t*-test, error bars represent ± SEM, *** indicate *P*<0.001. N = 4, each sample was measured in triplicate.

Following independent confirmation by Q-PCR of the STOX1A binding site in the SFRS7 promoter region (Fig. 1), the effect of STOX1A on SFRS7 transcription and translation was tested in SK-N-SH cells stably transfected with STOX1A or MOCK (negative control) constructs (Fig. 2A). Both SFRS7 mRNA (Fig. 2B) and protein levels (Fig. 2C) were found to be increased significantly and specifically upon STOX1A overexpression.

### STOX1A effectuates changes in 4R/3R tau ratio in SK-N-SH cells

Isoforms of the tau protein exhibit either three (3R) or four microtubule-binding repeats (4R) depending on alternative splicing of tau exon 10 [11]. As it has been shown that SFRS7 effects tau 4R splicing [9], [17], we argued that the increased SFRS7 transcript levels cause a change in tau 4R/3R ratios. Indeed, STOX1A leads to a significant increase in tau 4R/3R ratio (Fig. 2D).

**Figure 3:**
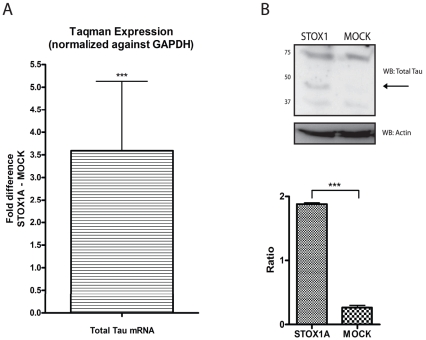
STOX1A upregulates total tau RNA and protein levels in stable transfected SK-N-SH cells. (A) Quantitative RT-PCR show a mean 3,59 fold (mean ΔΔCt −1,84) increased mRNA expression for total tau. Bars are mean ± SEM. P<0.05 (one sample t-test with theoretical mean 0). N = 4, each sample was measured in triplicate.(B, upper image) Expression of endogenous total tau protein was determined with a total tau specific antibody by western blot using total cell protein extracts obtained from stable STOX1A and MOCK transfected SK-N-SH cells. An antibody specific for actin was used as a loading control. Westernblot image is a representative of at least 3 independent experiments. (B, lower graph) Quantification of total tau protein was performed using densitometry. The ratio number of obtained band intensities for STOX1A divided by actin was compared to the ratio number of obtained band intensities for MOCK divided by actin for 3 independent experiments. A significant increase for the STOX1A ratio number was found compared to the MOCK ratio number. *P*-values were calculated using two-tailed unpaired *t*-test, error bars represent ± SEM, *** indicate *P*<0.001.

Secondly, endogenous total tau levels were also elevated, both at the mRNA (Fig. 3A) and protein levels (Fig. 3B).

### SFRS7-dependent tau exon 10 splicing by STOX1A is specific for glial cells

**Figure 4:**
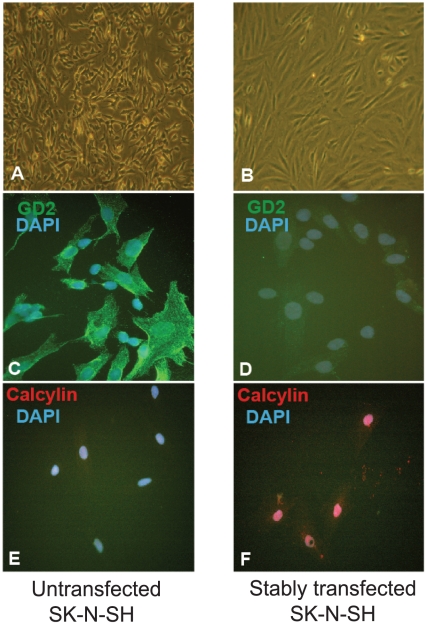
Stably transfected SK-N-SH cells consist of glial cells only. (A) Morphology of untransfected SK-N-SH cells or (B) stably transfected SK-N-SH cells. The G418 (Roche) antibiotic selection procedure for selection of positive STOX1A and MOCK stable transfected cells resulted by clonal selection in a homogeneous cell population that is composed of the glial (S-type) type. (C) Immunophenotype of untransfected SK-N-SH cells showing intense membrane staining for GD2 but lack nuclear calcylin staining (E). No GD2 membrane staining was observed in stably transfected SK-N-SH cells (D) while cells show high calcyclin nuclear staining (F). Green: GD2, Red: Calcyclin, Blue: DAPI.

**Figure 5:**
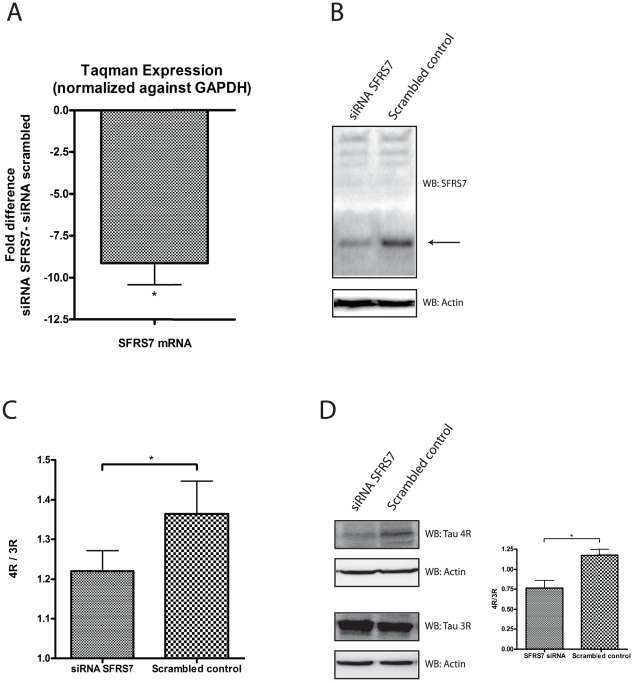
SFRS7 acts as a tau exon 10 splicing enhancer in the glial cell-line U- 373 MG. (A) Knockdown of endogenous SFRS7 protein was determined with quantitative RT-PCR showing a mean 9,13 fold (mean ΔΔCt 3.19) decreased endogenous mRNA expression for SFRS7. Bars are mean ± SEM.* indicate P<0.05 (one sample t-test with theoretical mean 0). N = 4, each sample was measured in triplicate. (B) Knockdown of endogenous SFRS7 protein was determined with a SFRS7 specific antibody by western blot using total cell protein extracts obtained from SFRS7 siRNA and scrambled transfected U-373 MG cells. An antibody specific for actin was used as a loading control. Westernblot image is a representative of at least 3 independent experiments. (C) Ratio numbers were obtained by dividing endogenous 4R and 3R tau mRNA concentrations from U373-MG cells transfected with siRNA SFRS7 or scrambled siRNA. Endogenous 4R and 3R mRNA concentrations were obtained as described in the material and methods section. A significant decrease of the 4R/3R tau ratio was seen for siRNA SFRS7 compare to scrambled siRNA transfected cells. *P*-values were calculated using two-tailed unpaired *t*-test, error bars represent ± SEM, * indicate *P*<0.05. N = 4, each sample was measured in triplicate. With the use of densitometry on obtained westernblot images (D) a similar significant decrease of the 4R/3R tau ratio was seen on a protein level (D, right graph). Significance was calculated by combining the densitometry data from 3 independent experiments. *P*-values were calculated using two-tailed unpaired *t*-test, error bars represent ± SEM, * indicate *P*<0.05

The cell line, SK-N-SH, we used for the above in-vitro experiments comprises a heterogeneous subpopulation of neuroblastic (N-type) and substrate-adherent (S-type) cells [12]–[15]. As we observed a change in morphology due to clonal selection following stable transfection (Fig. 4A vs 4B) we tested the possibility that the phenomenon we observed was cell-specific, i.e. either restricted to the neuroblastic (N-type) or glial (S-type) cell. Immunostaining for disialoganglioside (GD2) (Figs. 4C, –D) and calcyclin (Figs. 4E–F) showed the stably-transfected cells to consist of glial cells (S-type) only (calcyclin- positive, GD2-negative) [15]. Furthermore, no significant changes in SFRS7 expression and tau 4R/3R ratios were found upon stable transfection of STOX1A in the SK-N-SH N-type subclone cell- lineage SH-SY5Y (data not shown). Given this, and to independently confirm that the effect of STOX1A on exon 10 tau splicing via SFRS7 is glial cell-specific, we tested U-373 MG cells as a cellular model for glial cells. To confirm that in the STOX1A-SFRS7-tau splicing pathway, the effect of SFRS7 on tau splicing is direct in U-373 MG cells we performed siRNA knockdown experiments against SFRS7 in the untransfected U-373 MG cell-line and measured 3R and 4R tau concentrations for SFRS7 siRNA treated and control samples to calculate 4R/3R tau ratios. Significant SFRS7 knockdown was confirmed by measuring endogenous SFRS7 mRNA levels (Fig. 5A) and protein levels on western blot (Fig. 5B). Furthermore, a significant decrease in the 4R/3R ratio for SFRS7 siRNA treated cells was seen compared to the scrambled control (Fig. 5C) which was confirmed on western blot (Fig. 5D).

**Figure 6:**
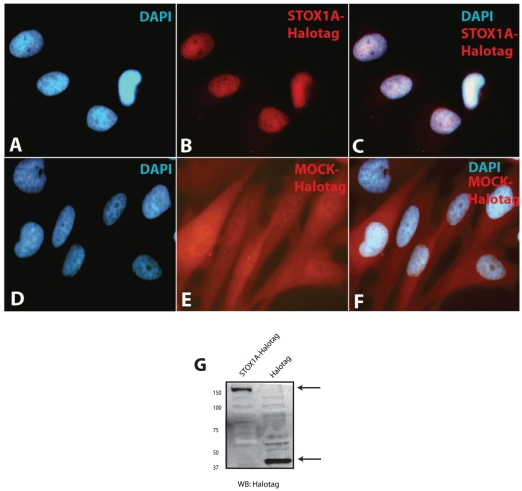
Expression analysis of STOX1A in stable transfected U-373 MG cells. (A, –C) Immunofluorescence shows primarily nuclear staining for STOX1A-Halotag protein in stable transfected U-373 MG cells while stable MOCK-Halotag transfected U-373 MG cells shows diffuse cytoplasmatic staining (D, –F). (G) Expression of STOX1A protein was determined with an anti-Halotag specific antibody by western blot using total cell protein extracts obtained from stable STOX1A (left lane) and MOCK (right lane) transfected U373 cells. A specific band representing STOX1A-halotag protein was observed at its expected size of 150 kd. Halotag (MOCK) protein was detected at its expected size of 34 kd. Westernblot image is a representative of at least 3 independent experiments.

Next, we stably transfected U-373 MG cells with STOX1A to determine the effect of STOX1A on tau exon 10 splicing via SFRS7. First, we characterized the STOX1A expression pattern in U-373 MG cells using immunofluorescence which showed strong nuclear (Fig. 6A, –C) staining for STOX1A protein. Furthermore, expression of STOX1A was confirmed on westernblot (Fig. 6G).

**Figure 7:**
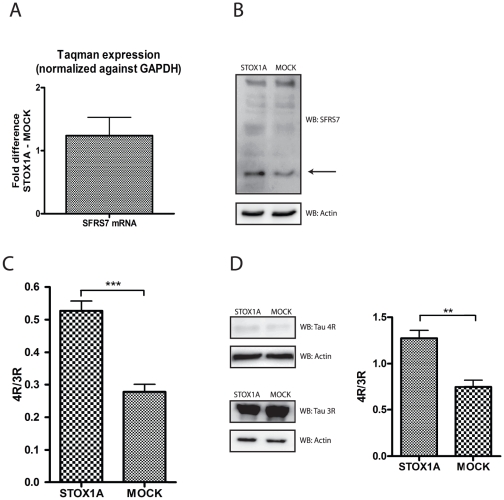
Tau exon 10 splicing is increased upon stable STOX1A overexpression via SFRS7 in U-373 MG cells. (A) Quantitative RT-PCR shows a mean 1,24 fold (mean ΔΔCt is −0,306) increased mRNA expression for SFRS7. Bars are mean ± SEM. * indicate *P*<0.05 (one sample t-test with theoretical mean 0). N = 4, each sample was measured in triplicate. (B) Expression of endogenous SFRS7 protein was determined with a SFRS7 specific antibody by western blot using total cell protein extracts obtained from stable STOX1A and MOCK transfected U-373 MG cells. Westernblot image is a representative of at least 3 independent experiments. (C) Ratio numbers were obtained by dividing endogenous 4R and 3R tau mRNA concentrations for U373-MG cells stably transfected with STOX1A or MOCK constructs. Endogenous 4R and 3R mRNA concentrations were obtained as described in the material and methods section. A highly significant increase of the 4R/3R tau ratio was seen for STOX1A compare to MOCK transfected cells. *P*-values were calculated using two-tailed unpaired *t*-test, error bars represent ± SEM, *** indicate *P*<0.001. N = 4, each sample was measured in triplicate. With the use of densitometry on obtained westernblot images (D) a similar significant decrease of the 4R/3R tau ratio was seen on a protein level. Significance was calculated by combining the densitometry data from 3 independent experiments. *P*-values were calculated using two-tailed unpaired *t*-test, error bars represent ± SEM, * indicate *P*<0.05

Secondly, we determined the effect of stable STOX1A expression on SFRS7 expression and tau exon 10 splicing. Identical effects on SFRS7 mRNA (Fig. 7A) and protein (Fig. 7B) levels were seen as for the SK-N-SH (S-type) cells (Fig. 2). Furthermore, 4R/3R tau ratio's (Fig. 7C) were significantly increased which was confirmed on western blot (Fig. 7D) thereby showing the effect of STOX1A on tau processing via SFRS7 in glial cells.

**Figure 8:**
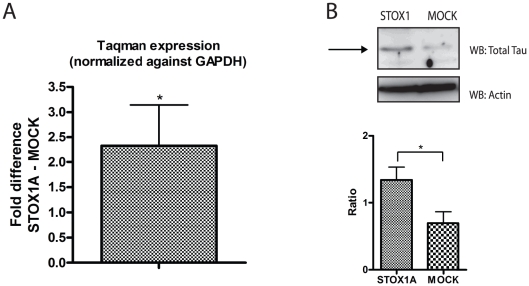
STOX1A effectuates endogenous total tau expression in U-373 MG cells. (A) Quantitative RT-PCR show a mean 2,33 fold (mean ΔΔCt is −1,22) increased mRNA expression for total tau. Bars are mean ± SEM. * indicate *P*<0.05 (one sample t-test with theoretical mean 0). N = 4, each sample was measured in triplicate. (B) Expression of endogenous total tau protein was determined with a total tau specific antibody by western blot using total cell protein extracts obtained from STOX1A and MOCK transfected U-373 MG cells. An antibody specific for actin was used as a loading control. Westernblot image is a representative of at least 3 independent experiments. (B, lower graph) Quantification of total tau protein was performed using densitometry. The ratio number of obtained band intensities for STOX1A divided by actin was compared to the ratio number of obtained band intensities for MOCK divided by actin for 3 independent experiments. A significant increase for the STOX1A ratio number was found compared to the MOCK ratio number. *P*-values were calculated using two-tailed unpaired *t*-test, error bars represent ± SEM, *** indicate *P*<0.001.

Finally, as for the SK-N-SH (S-type) cells (Fig. 3), significantly increased endogenous total tau expression, both at the mRNA (Fig. 8A) and protein levels (Fig. 8B), was seen upon stable STOX1A overexpression in the U-373 MG cell-line.

## Discussion

Here we describe the genome-wide exploration of genomic regions bound by STOX1A in the neuroblastoma cell-line SK-N-SH. For this, an antibody-free chromatin immunoprecipitation procedure was used followed by massively parallel sequencing. Of the genes bound, the SFRS7 gene was selected for in-depth functional analysis. We found that SFRS7-mediated splicing of tau exon 10 is directly regulated by STOX1A in glial cells. Previously we showed the effect of STOX1A on the amyloid pathway in neuronal SK-N-SH cells [4]. Here we show the effect of STOX1A on both total tau expression as well as tau processing (exon 10 splicing) in glial cells. The effect of STOX1A on tau expression and processing additionally confirms the role of this transcription factor in central pathways underlying neurodegeneration including AD.

The data on tau splicing appear initially in conflict with the observation made by Gao and co-workers who found that SFRS7 acts as a tau exon 10 splicing silencer in SK-N-SH cells thereby decreasing the ratio of 4R/3R tau [9]. Our results show the reverse. However, we cannot exclude that Gao and co-workers used a mixed SK-N-SH cell population consisting of primarily N-type and little S-type cells, while ours consisted of the S-type subpopulation only. Possibly, in our situation, the S-type cells transfected more easily. Therefore, transfected S-type cells were able to overgrow the N-type cells which died from the neomycin selection. This resulted in a cell population consisting of the S-type subpopulation only.

Furthermore, while our results indicate that SFRS7 acts as a splicing enhancer in the S-type of cells, the effect of STOX1A on SFRS7 is also restricted to the S-type cells whereas we could not find any significant changes in SFRS7 expression upon stable STOX1A expression in the N-type subclone cell line SH-SY5Y (data not shown). This does not exclude the role of SFRS7 acting as a tau exon 10 splicing silencer in the neuronal S-type cells, in fact, SFRS7 has been shown to suppress tau exon 10 inclusions in the neuroblastoma cell-line SH-SY5Y by Ding *et al*. [17]. Given this, we hypothesize that both the function of SFRS7 acting as a splicing enhancer or silencer, and the effect of STOX1A on SFRS7 expression is cell-type dependent (neuronal versus glial). Therefore our aim was to further investigate the function of STOX1A on SFRS7 in glial cells where SFRS7 acts as an exon 10 splicing enhancer. This theory is supported by our finding that knockdown of SFRS7 in the U-373 MG cell-line shows an decrease in the 4R/3R tau ratio while stable overexpression of STOX1A, which induces SFRS7 expression, increases the 4R/3R tau ratio.

The opposite effect on tau exon 10 splicing seen after SFRS7 downregulation, as shown for glial cells in this paper, supports the cell type dependent regulation of this splicing factor which was also observed by Ding and co-workers [17]. Possibly, SFRS7 acts as a tau exon 10 splicing enhancer in glial cells by binding to yet to discovered exonic and intronic enhancers, which would not be surprising because it has been shown that SFRS7 mainly acts as a splicing enhancer, instead of silencer as shown by Gao and co-workers [17]–[20].

In LOAD, neurofibrillary tangles are one of the main hallmarks [21]. While we previously found that in advanced stages of LOAD (Braak 3–6), STOX1A primarily stains positive in hippocampal neurons with neurofibrillary tangles (but also microglia) [4], the implication in LOAD with our presented findings for STOX1A in glial cells would therefore be possibly limited.

However, while STOX1A expression is found primarily in the brain and dysregulation of the balance between glial 4R/3R tau ratio by a selective increase in either 3R-tau or 4R-tau is commonly seen in a range of diverse neurodegenerative diseases other than LOAD, including frontotemporal dementia with Parkinsonism linked to chromosome 17 (FTDP-17), Pick's disease (PD), progressive supranuclear palsy (PSP), and corticobasal degeneration [22], further exploration of where STOX1A and/or SFRS7 expression are found in these neurodegenerative diseases would yield great insight of their involvement.

Interestingly, independently of SFRS7 transactivation by STOX1A we also observed a significant increase in total tau mRNA and protein levels in both SK-N-SH (S-type) and U-373 MG cells. While this effect is not seen after knockdown of SFRS7, this effect is also STOX1A-dependent. As no binding site was identified for STOX1A in the tau promoter (Dataset S1), it would therefore be interesting to further investigate which gene, other than tau or SFRS7 but bound by STOX1A, controls total tau expression in glial cells.

Although we selected a single gene for functional analysis, combined or follow-up analysis of the other genes identified will likely generate novel results providing and permitting mechanistic insight in pathways important for e.g. neurodegeneration. The antibody-free ChIP procedure can be applied to other transcription factors and cellular systems as well and is highly informative when combined with massively parallel sequencing as done in the present paper.

In conclusion, while previous findings by van Dijk and co-workers have shown a functional link between STOX1A and LOAD, we here provide further evidence that STOX1A has important functions in the pathways leading to neurodegeneration.

## Materials and Methods

### Cell culture and transfection

SK-N-SH human neuroblastoma cells were obtained from the American Type Culture Collection (ATCC, Manassas, VA). All reagents for cell culture were purchased from Invitrogen Life Technologies, Inc. (Burlington, Canada). SK-N-SH and U-373 MG cells were cultured at 37°C in a humidified atmosphere of 5% CO_2_ and 95% O_2_ in Iscove's Modified Dulbecco's Medium (IMDM) supplemented with 10% fetal calf serum and 100 U/ml penicillin, and 100 g/ml streptomycin. Cells were subcultured in medium every 2–3 days following harvesting by trypsinization (HBSS containing 5% trypsin). The ORF (Open Reading Frame) of the STOX1A gene was subcloned into the Halotag pF5K neomycin CMV Flexi vector according to the manufacturers protocol (Promega). For transfection the calcium phosphate method was used [16]. Briefly, at the time of transfection, cells were at 70% confluence. By vortexing 2X HEBS (HEPES-buffered saline) with a solution of 2.5 M CaCl_2_ and 20 µg of plasmid DNA a co-precipitate of DNA and CaPO_4_ was allowed to form. After incubation for 30 min at room temperature, the precipitate was added to the cells and the medium was changed after 24 h. Cells were used 48 h post-transfection for further analysis.

For stable transfections SK-N-SH or U-373 MG cells were transfected either with the pF5K-STOX1A or empty pF5K constructs. Selection of positive clones was possible due to the presence of the neomycin resistant gene present in the constructs. Positive clones were maintained in complete IMDB medium supplemented with 800 µg/ml G418 (Roche) until reaching confluence and subcultured every 2–3 days for about 4 weeks. A population of positive clones was harvested for further analysis. As confirmed with qRT-PCR (See below) using TaqMan probes (Applied Biosystems) for STOX1A, two stable SK-N-SH cell-lines which over-expressed STOX1A 25 and 30 fold, and 6 stable U-373 MG cell-lines with a mean 64 fold over-expression of STOX1A were selected. Protein expression of the STOX1A-Halotag proteins was confirmed by western blot. Additionally, plasmid DNA quantification using qPCR (see below) with primers and probe specific for the constructs backbone showed similar copy numbers in each cell-line.

Knockdown of endogenous SFRS7 was performed by transfecting siRNA's against SFRS7 and control siRNA (Santa Cruz) in untransfected U-373 MG cells with Lipofectamine™ RNAiMAX (Invitrogen) according to the manufactures protocol. Cells were harvested 48 hours post-transfection and RNA was isolated as described in the Quantitative PCR and RT-PCR section.

### Chromatin immunoprecipitation

Transfected cells were treated with formaldehyde to create protein-DNA crosslinks. Cytoplasmatic lysis was performed to reduce competition of cytoplasmatic STOX1A-Halotag proteins against nuclear STOX1A-Halotag proteins with the Halotag resin. Nuclear lysate was subsequently fragmented by sonication. The nuclear lysates were split into two equal parts of which one was treated with Halotag blocking ligand to function as a negative control. Both the samples and controls were treated using Halotag resin according to the Halo-ChIP system protocol in the presence of proteinase inhibitors. After reversal of crosslinks the DNA was purified using the Qiaquick PCR purification kit (Qiagen). The ChIP-Seq method can be found in the additional material section (**Text S1**).

### Quantitative PCR and RT-PCR

Standard quantitative PCR was performed on an ABI7300 (Applied Biosystems) using probes (5′-FAM 3′-TAMRA labeled SFRS7 probe 5′-CGCCCAGGGCTCGA GTGAC-3′) and primers (SFRS7 forward: 5′-ACGCGACATGATGACAGAC- 3′, SFRS7 reversed: 5′-CGCATATATAAACGCGAACC-3′) specific for the SFRS7 region obtained in the ChIP-Seq experiments in the presence of 1M betaine and ROX reference dye, and corrected for input using the non-intron-spanning Glyceraldehyde 3-phosphate dehydrogenase (GAPDH) gene expression assay (Applied Biosystems). Input ChIP DNA was obtained from at least five independent ChIP experiments.

RNA isolation from transfected cells was performed using the RNeasy kit (Qiagen) including on-column DNase treatment. Quantitative RT-PCR using gene expression assays (Applied Biosystems) for SFRS7 and total tau was performed on an ABI7300. In addition, transfection effiencies were corrected by plasmid DNA quantification using the pF5 CMV-neo Flexi® Vector backbone present in all plasmids (pF5 forward: 5′-GCTTCGAGCAGACATGATAAG-3′, pF5 reversed: 5′-AGCAATAGCATCACAAA TTTCA-3′, 5′-FAM 3′-TAMRA labeled pF5 probe: 5′-TGGACAAACCACAACT AGAATGCAGT-3′). Data were obtained from at least four transfections from which each RNA sample was measured in triplicate.

### Western blot

Protein lysates from stable transfected cells were obtained by directly scraping cells into loading Buffer including *β-*mercaptoethanol. Lysates were separated by SDS-polyacrylamide gel electrophoresis, and electroblotted onto a PVDF-membrane. An antibody recognizing the Halotag protein (Promega) was used in combination with goat anti-rabbit horseradish peroxidase-conjugated secondary antibody (DAKO). To detect endogenous levels of SFRS7 and tau, SFRS7 antibody (9G8, Santa Cruz) or total tau antibody (Abcam) was used in combination with donkey anti-goat IgG horseradish peroxidase-conjugated secondary antibody (Santa Cruz) and goat anti-rabbit horseradish peroxidase-conjugated secondary antibody (DAKO), respectively. Mouse monoclonal antibodies specific for the tau 3R (RD3, clone 8E6/C11) or 4R (RD4, clone 1E1/A6) splice variants were obtained from Millipore and used in combination with goat anti-mouse horseradish peroxidase-conjugated secondary antibody (DAKO). Endogenous actin used for loading controls was detected with mouse monoclonal anti-actin antibody (Sigma Aldrich) used in combination with goat anti-mouse horseradish peroxidase-conjugated secondary antibody (DAKO). Protein bands were detected by an enhanced-chemiluminescence assay (GE Healthcare) on a LAS3000.

### Immunofluorescence

For immunofluorescence, untransfected and stable transfected SK-N-SH cells were grown on glass coverslips. Coverslips were fixed in 4% (PFA) paraformaldehyde for 15 min at room temperature. After fixation, coverslips were rinsed in PBS, 0.1% Triton X-100 and incubated with 1% Triton X-100 in PBS for 15 min at room temperature for permeabilization. Coverslips were washed in wash buffer (PBS, 0.1% Triton X-100, 2% BSA (Bovine serum albumin)) blocked with PBS, 0.1% Triton X-100, 5% BSA for 1 h and incubated with Calcyclin (Santa Cruz biotechnology) or GD2 (BD Pharmingen) antibody at 4°C overnight.

After washing with wash buffer, cells were incubated for 1 hour with the appropriate secondary antibodies conjugated with Alexa Fluor 488 or Alexa Fluor 568 (Invitrogen), washed with washing buffer and mounted with vectashield mounting solution containing DAPI for DNA counterstaining (Vector Laboratories).

For STOX1A-Halotag immunofluorescence, stable transfected cells grown on coverslips were incubated with TMR-Halotag ligand according the manufactures instructions (Promega). Coverslips were fixed in 4% (PFA) paraformaldehyde for 15 min at room temperature. After fixation, coverslips were washed with washing buffer and mounted with vectashield mounting solution containing DAPI for DNA counterstaining (Vector Laboratories).

### Tau splicing Assay and ratio calculation

qRT-PCR was performed with RNA isolated from the stable STOX1A transfected SK-N-SH and U-373 MG cell-lines and transiently transfected SFRS7 and scrambled siRNA U-373 MG cells on a Lightcycler 480 (Roche) using the SYBR Green master kit and primers specific for the 3R (3R-forward: 5′-GGAAGGTGCAAATAGTC-3′, 3R-reversed: 5′-CGACTGGACTCTGTCCTTGA-3′) or 4R tau (4R-forward: 5′-CGGGAAGGTGCAGATAATTAA-3′, 4R-reversed: 5′-GCCTAATGAGCCACACTT GGAG-3′) isoforms. Due to the high degree of homology between 3R and 4R isoforms, the 3R 5′-3′ primer has 4 Locked Nucleic Acid (LNA) modifications to ensure specificity. cDNA was synthesized with the SuperScript III Reverse Transcriptase kit (Invitrogen).Constructs containing the 3R or 4R tau isoforms with known DNA concentrations were used for the generation of a standard curve (concentrations for the constructs containing the 3R isoform were 10^−2^, 10^−3^, 10^−4^, 10^−5^ and 10^−6^ ng/µl and for the constructs containing the 4R isoform 10^−3^, 10^−4^, 10^−5^, 10^−6^ and 10^−7^ ng/µl). Experimental samples were measured and concentrations calculated by comparison to the standard curve. Tau ratios were calculated by dividing the mean 4R tau concentrations by the mean 3R tau concentrations of the treated samples. The same was done for the control treated samples. An unpaired t-test was used to calculate significance between the treated 4R/3R tau ratio and control treated 4R/3R tau ratio. Error bars represent ± SEM.

### Data analysis

For Real-time PCR data, a threshold cycle number, Ct, was measured as the PCR cycle at which the amount of amplified target reaches the threshold value. Quantification was determined by the 2^-ΔΔCt^ method as described in Applied Biosystems “Guide to Performing Relative Quantitation of Gene Expression Using Real-Time Quantitative PCR”, Section VII, Relative Quantitation of Gene Expression Experimental Design and Analysis http://www3.appliedbiosystems.com/cms/groups/mcb_support/documents/generaldocuments/cms_042380.pdf).Statistical analysis of the obtained data was carried out with the GraphPad Prism program.

For quantification of westernblots densitometry was used. Westernblot pictures were analysed with the software ImageJ according to the tutorial described on the website: http://lukemiller.org/index.php/2010/11/analyzing-gels-and-western-blots-with-image-j/. Statistical analysis on the obtained density values was carried out with Microsoft Excell and the GraphPad Prism program.

## Supporting Information

Dataset S1 **BED file as outputted by QuEST.** The BED file provided can be directly uploaded into the UCSC browser (http://genome.ucsc.edu/) as a custom track. The human assembly (Mar. 2006 NCBI36/hg 18) was selected as reference genome. Custom tracks show genomic regions in blue and potential binding peaks in red as calculated by QuEST.
(BED)

Dataset S2 **Regions (BED file, Dataset S1) of functional importance generated by CEAS as RefSeq genes.** Can be opened in Microsoft Excell.
(XLSX)

Text S1 **Supplementary ChIP sequencing methods.** Can be opened in Microsoft Word.
(DOC)
