# Supplementary material for: SFRS7-Mediated Splicing of Tau Exon 10 Is Directly Regulated by STOX1A in Glial Cells
Source: PLoS One. 2011 Jul 6;6(7):e21994. doi: 10.1371/journal.pone.0021994 (PMC3130792; doi:10.1371/journal.pone.0021994)
Supplement: Text S1 — Supplementary ChIP sequencing methods. Can be opened in Microsoft Word. (DOC) [file pone.0021994.s003.doc]

**Additional file 3: Supplementary ChIP sequencing methods**

**Massively Parallel Genomic Sequencing**

Multiple samples of precipitated HaloChIP DNA were validated with QPCR on enrichment with a confirmed STOX1 bound region found in a previously performed ChIP-shotgun cloning procedure (Data not shown). Enriched samples were pooled and used for high-throughput sequencing (ChIP-Seq) on a Genome Analyzer IIx (Illumina).

10–80 ng of pooled HaloChIP DNA was used for DNA library construction by the Genomic DNA Sample Preparation Kit (Illumina) following the manufacturer’s protocol of sample preparation for ChIP-Sequencing of DNA. Enriched adapter-ligated DNA fragments in the range of 200–350 bp were size selected using 2% agarose electrophoresis. The selected DNA libraries were then additionally amplified using a 15-cycle PCR. The adapter ligated DNA was purified directly using spin columns provided in a QIAquick PCR purification kit (Qiagen).

**Sequence Alignment**

Single-read sequencing for 36 cycles was performed. All 36-bp sequence reads were aligned to the repeat-masked human genomic reference sequence (NCBI Build 36, version 48) downloaded from the Ensembl Genome Browser (http://www.ensembl.org), using the ELAND program in the GAPipeline-1.4.0 software package provided by Illumina. A result output file was generated after running ELAND.

**ChIPseq peak calling**

For peak calling we used the ELAND output files in the program QuEST 2.4. Firstly, ChIP default parameters were selected for a transcription factor with defined motif and narrow binding site (bandwidth = 30 bp and region size = 300 bp). Stringent peak calling parameters were then selected as recommended by QuEST (ChIP threshold = 0.164, Enrichment fold = 3 and Rescue fold = 3).

Each peak was ranked according to a Q-value produced from the *p*-values calculated by Benjamini correction in QuEST. This ranking indicated the certainty of enrichment between the experimental versus control data on a genome-wide level.
